# Design and Simulation of InGaN-Based Red Vertical-Cavity Surface-Emitting Lasers

**DOI:** 10.3390/mi15010087

**Published:** 2023-12-30

**Authors:** Tai-Cheng Yu, Wei-Ta Huang, Hsiang-Chen Wang, An-Ping Chiu, Chih-Hsiang Kou, Kuo-Bin Hong, Shu-Wei Chang, Chi-Wai Chow, Hao-Chung Kuo

**Affiliations:** 1 Department of Photonics, College of Electrical and Computer Engineering, National Yang Ming Chiao Tung University, Hsinchu 300093, Taiwan; 2 Semiconductor Research Center, Hon Hai Research Institute, Taipei 114699, Taiwan; 3 Department of Foreign Languages and Literatures, College of Liberal Art, National Taiwan University, Taipei 106310, Taiwan; 4 Research Center for Applied Sciences, Academia Sinica, Taipei 115201, Taiwan

**Keywords:** InGaN, vertical-cavity surface-emitting laser, nanoporous DBR, high-index contrast grating, staggered MQW

## Abstract

We propose a highly polarized vertical-cavity surface-emitting laser (VCSEL) consisting of staggered InGaN multiple quantum wells (MQWs), with the resonance cavity and polarization enabled by a bottom nanoporous (NP) n-GaN distributed Bragg reflectors (DBRs), and top TiO_2_ high-index contrast gratings (HCGs). Optoelectronic simulations of the 612 nm VCSEL were systematically and numerically investigated. First, we investigated the influences of the NP DBR and HCG geometries on the optical reflectivity. Our results indicate that when there are more than 17 pairs of NP GaN DBRs with 60% air voids, the reflectance can be higher than 99.7%. Furthermore, the zeroth-order reflectivity decreases rapidly when the HCG’s period exceeds 518 nm. The optimal ratios of width-to-period (52.86 ± 1.5%) and height-to-period (35.35 ± 0.14%) were identified. The staggered MQW design also resulted in a relatively small blue shift of 5.44 nm in the emission wavelength under a high driving current. Lastly, we investigated the cavity mode wavelength and optical threshold gain of the VCSEL with a finite size of HCG. A large threshold gain difference of approximately 67.4–74% between the 0th and 1st order transverse modes can be obtained. The simulation results in this work provide a guideline for designing red VCSELs with high brightness and efficiency.

## 1. Introduction

In recent decades, vertical-cavity surface-emitting lasers (VCSELs) have emerged as a distinct semiconductor laser that emits light vertically from its top surface, setting it apart from edge-emitting lasers (EELs). The distinctive structure design of VCSEL has captured the interest of experts and scholars due to its numerous advantages, including vertical beam emission, circular spot and low divergence angle, low threshold operation, compact size, and high output power enabled by the laser array.

Thus, VCSELs have been widely utilized as light sources across various fields such as laser display [1,2], optical communication [3,4,5], data storage [6,7], gas sensors [8,9], augmented reality (AR) and virtual reality (VR) displays [10,11], as well as three-dimensional (3D) image sensing and light detection and ranging (LiDAR) applications within the visible to mid-infrared (MIR) band [12,13,14,15,16]. Recently, there has been rapid experimental development in InGaN-based vertical-cavity surface-emitting lasers to achieve visible light wavelength light sources [17,18,19]. Moreover, research reports indicate that the wavelength range of vertical-cavity surface-emitting lasers can be extended to the ultraviolet (UV) range [20,21]. It is well known that the vertical-cavity surface-emitting laser structure is commonly constructed by sandwiching an optically active region that provides optical gain between two high-reflectivity cavity mirrors. Typically, these critical components comprise multilayer structures known as multiple quantum wells (MQWs) and distributed Bragg reflectors (DBRs).

Notably, laser cavity mirrors with high reflectivity are essential for proper laser operation. As a result, several types of distributed Bragg reflectors have been developed, including epitaxial Al_0.12_Ga_0.88_As/Al_0.9_Ga_0.1_As DBRs [22], GaN/AlGaN DBRs [23,24], dielectric Ta_2_O_5_/SiO_2_ DBRs [25,26], and SiO_2_/TiO_2_ DBRs [27]. In recent years, nanoporous (NP) n-doped GaN DBRs have garnered significant attention for their ability to enhance the light extraction efficiency of InGaN-based light-emitting diode (LED). These nanoporous mirrors can be easily constituted with high reflectivity and directly grown on the LED and laser wafers. They can be developed through chemical etching to fabricate blue VCSELs [28,29] and red LEDs [30]. Additionally, high-index contrast gratings (HCGs) have also been widely utilized in the development of VCSELs, such as mid-infrared VCSELs composed with air-suspended InP HCGs [31,32], GaAs-based near-infrared VCSELs integrated with monolithic GaAs or AlGaAs HCGs [33,34]. Additionally, the disclosures of TiO_2_ HCGs [19,35] and monolithic GaN HCGs [36] for constituting InGaN-based blue VCSELs. The excellent advantages of thickness reduction and precise polarization selectivity drive these developments. Previous research has proposed using monolithic high-refractive-index contrast gratings integrated with metal contacts, known as semiconductor-metal subwavelength gratings (SMSG), which can serve as optical couplers and current injectors in VCSELs. To optimize the performance of these SMSGs, a plane wave admittance method algorithm was employed to search for the local maxima of power reflectance (LMPR) by varying the geometrical parameters such as the period, duty cycle, and height of the SMSG. The SMSG can comprise various materials such as GaN, GaAs, AlGaAs, InP, and Si. This report provides the suitable size of GaN SMSG for the 470 nm blue light and 540 nm green light wavelengths [37].

It is noteworthy that prior research has consistently evidenced a blue shift in the emission wavelengths of InGaN-based blue/yellow/red light-emitting diodes. Furthermore, the external quantum efficiency (EQE) of these LEDs is found to decrease, while the full width at half maximum (FWHM) of their emission spectra shows an increase [38,39,40]. This phenomenon can be attributed to an InGaN quantum well with a higher indium content, leading to a strong piezoelectric field-induced quantum-confined Stark effect (QCSE). Additionally, the strong QCSE can be mitigated by the high injection of carriers when LEDs operate at high current densities. Furthermore, previous research has indicated that blue and green LEDs with staggered InGaN multiple quantum wells can significantly enhance radiative emission efficiency by increasing the spatial overlap of electron–hole wave functions in the MQWs and reducing band bending [41,42]. Moreover, a previous study demonstrated that the QCSE can be minimized by incorporating quantum barriers with specific compositions of InGaN. These quantum barriers are designed to generate polarization-induced bulk charges, as determined through numerical simulations [43,44].

Interestingly, the staggered MQW structure design has recently been demonstrated in InGaN-based blue LEDs [45], green LEDs [46], and AlGaN-based deep UV lasers [47]. Additionally, the optoelectronic characteristics of InGaN-based green micro-resonant cavity light-emitting diodes (µ-RCLEDs), which consist of a three-layer staggered InGaN MQWs, bottom nanoporous n-GaN DBRs, and top Ta_2_O_5_/SiO_2_ DBRs, have been numerically investigated [48]. However, no literature has discussed the design of InGaN-based red vertical-cavity surface-emitting lasers using staggered MQWs, nanoporous DBRs, and high-index contrast grating mirrors.

This paper proposes and investigates a novel InGaN-based red VCSEL structure consisting of NP n-doped GaN/undoped GaN DBR, staggered MQW structure, and TiO_2_ high-index contrast grating. We conducted a comprehensive simulation-based investigation to analyze the optical reflectivity, cavity mode wavelengths, and optical threshold gains by varying the geometric parameters of NP GaN DBR and TiO_2_ HCG. At the same time, the emission spectrum of staggered InGaN MQWs for various current injections was also discussed. Finally, the impact of the TiO_2_ HCG pattern size on the wavelengths of the cavity modes and the threshold gains will be thoroughly investigated and explained. This will significantly contribute to developing innovative red-light sources with high polarization and luminance.

## 2. Simulation Method

**Figure 1:**
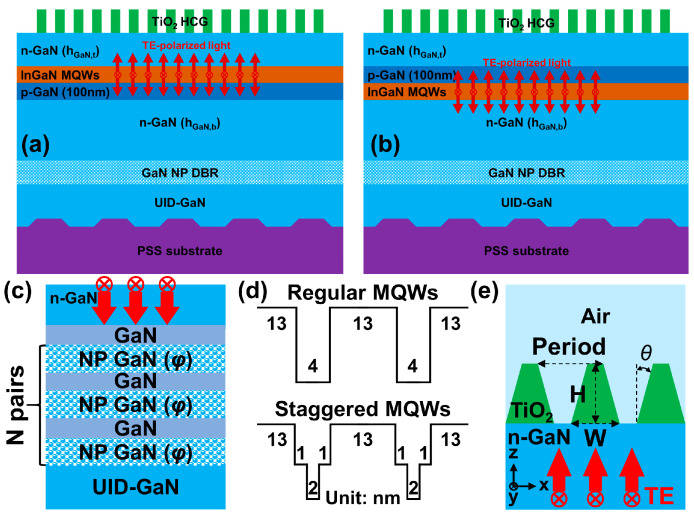
Schematic drawings of proposed InGaN-based red VCSELs with nanoporous (NP) GaN DBR and TiO_2_ HCG for (**a**) simulation sample 1: n-p-i-n structure design and (**b**) simulation sample 2: n-i-p-n structure design. The simulation model sketches of (**c**) NP GaN DBR, (**d**) regular and staggered MQWs, the numbers 13, 4, 2 and 1 represent the thickness of each InGaN layer, and (**e**) TiO_2_ HCG deposited on the n-GaN layer. The red arrows in (**a**–**c**,**e**) indicate the simplified diagrams of incident lights with TE polarization.

This section introduces our proposed InGaN-based red vertical-cavity surface-emitting lasers with a symmetric staggered active region design, top TiO_2_ HCG mirror, and bottom nanoporous GaN distributed Bragg reflectors. The schematic drawings of two laser structures for numerical simulations are presented in Figure 1a,b, referred to as simulation sample 1 (n-p-i-n structure) and sample 2 (n-i-p-n structure). According to the existing experience, the proposed laser structures can be grown on a continuous, high-quality surface formed by an unintentionally doped (UID) GaN layer and a patterned sapphire substrate (PSS). Subsequently, the nanoporous n-doped GaN/undoped GaN multilayers were fabricated using a chemical etching process. For simplification, the simulation domain does not include the patterned sapphire substrate. On the other hand, in recent developments, InGaN-based LEDs with a current injection structure have been proposed and demonstrated, indicating that carriers from the p-n junction diffuse to the active region outside the p-n junction [49,50]. These works contribute a potential solution to mitigate the efficiency degradation of InGaN-based LEDs.

To increase carrier injection density and inhibit quantum efficiency degradation of InGaN-based red VCSELs, the p-n junction was also considered in our designed structure. Including a p-n junction can eliminate the necessity of wafer bonding and flip-chip processes during fabrication. Additionally, the simulation model sketches of the regular and staggered InGaN MQW structures and high-reflectivity mirrors for the proposed red VCSELs are depicted in Figure 1. We will numerically discuss the current-driven emission spectrum for the comparison of regular MQW (13 nm GaN/4 nm InGaN/13 nm GaN) design and staggered MQW (13 nm GaN/1 nm InGaN/2 nm InGaN/1 nm InGaN/13 nm GaN) design. The diameter of the current injection area was set to be 10 μm, and these numerical calculations were conducted using the PICS3D 2023 software package.

In the proposed InGaN-based red VCSELs, key structural variables used in the simulations for the design of a 612 nm transverse electric (TE) polarized surface-emitting laser involves the pair number and air voids ratio (*φ*) in the NP GaN DBR, the sidewall angle (*θ*), height (H), and width (W) in the TiO_2_ HCG. In this study, to address the unreliability caused by the random distribution of pores in NP-GaN DBR, we approached the DBR as a homogeneous material with a specific refractive index. This method effectively removes the dependency on the size of the simulation region. We calculated the refractive index of NP-GaN DBR using the following equation, $n_{NP-GaN}^{2}=\varphi \cdot n_{Air}^{2}+(1-\varphi )n_{GaN}^{2}$, also known as the air voids ratio, thereby ensuring more consistent and size-independent results in our simulations. To design a high reflectivity DBR, the NP GaN and GaN layer have a quarter-wave thickness, that is, $\lambda /\left(4n_{NP-GaN}\right)$. In cases φ=0.2~0.8, the thicknesses (refractive index) of NP GaN and GaN used in the simulations are 70.8~110.5 nm (2.26~1.87) and 64.8 nm (2.36), respectively. The simulation optimization for TE polarization reflectivity was calculated using the finite element software, COMSOL 6.1. TE polarization means the electric field is parallel to the y-axis, as shown in Figure 1e. The p-GaN layer’s thickness is 100 nm, while the top and bottom n-doped GaN layers are defined as h_GaN,t_ and h_GaN,b_, respectively. These thicknesses of n-GaN layers are crucial for assessing cavity wavelength shifts and optical threshold gains, and they are an integral part of the VCSEL’s performance optimization.

## 3. Results and Discussion

This section will be divided into five subsections to provide concise descriptions and interpretations of optoelectronic simulation results for designing the InGaN-based red VCSELs emitting a TE-polarized light. These five subsections will cover (1) the optical reflectivities of NP GaN DBR changing with pair number and air voids ratio, (2) the optical reflectivities of TiO_2_ HCG varying with grating’s width, height and period, (3) the emission spectrum and emission peak wavelength shifts of regular and staggered InGaN MQW structure designs, (4) n-GaN thickness-dependent laser cavity mode wavelengths and optical threshold gains calculated by a VCSEL unit cell model, and (5) the impacts of the period number of TiO_2_ grating on lasing wavelengths and thresholds calculated by a 2D finite VCSEL model.

### 3.1. Selection of Nanoporous GaN DBRs

In this subsection, the bottom mirror for our proposed red VCSEL is constructed using nanoporous n-doped GaN/undoped GaN DBRs. To investigate the influences of air voids ratio, the pair number of NP GaN DBRs, and light incident angle on the optical reflectivity.

**Figure 2:**
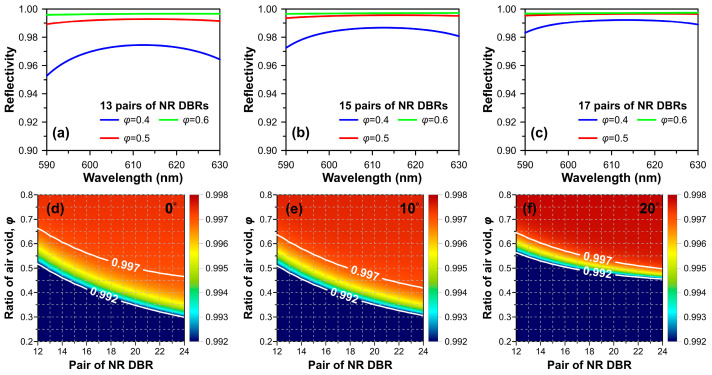
Wavelength-dependent reflectivities of (**a**) 13, (**b**) 15, and (**c**) 17 pairs of NP GaN DBRs for three ratios of air voids. Reflectivity maps were calculated as functions of pair number and ratio of air voids of NP GaN DBR for light incident angles of (**d**) 0°, (**e**) 10°, and (**f**) 20°. The white lines plotted in (**d**–**f**) indicate the high-reflectivity band between 0.992 and 0.997.

To prevent the overestimation of reflective capability, we assumed an absorption loss of 30 cm^−1^ inside the NP n-doped GaN induced by free-carrier absorption and irregularly distributed air voids for simplification. The reflectivity spectra for 13, 15, and 17 pairs of NP GaN DBRs under normal incidence for three kinds of air voids ratios, *φ* = 0.4, *φ* = 0.5, and *φ* = 0.6, were calculated, as shown in Figure 2a–c. The simulation results indicate that 13 pairs of NP DBR with an air voids ratio of *φ* = 0.5 can achieve a 0.99 reflectivity over a broad wavelength range of 610 ± 20 nm. Furthermore, when the pair number of NP GaN DBR exceeds 17, the difference in calculated reflectivity caused by the ratio of air voids appears to be negligible, with a discrepancy value of approximately 0.001.

The subsequent color maps and white contours depict the calculated optical reflectivity as functions of pair number and air voids ratio of NP GaN DBR for a light wavelength of 612 nm and TE-polarized light with incident angles of 0°, 10°, and 20°, are shown in Figure 2d–f. The white lines plotted in these figures indicate the high-reflectivity band between 0.992 and 0.997. The lower-left area of the calculated TE-polarized reflectivity map shows the optical reflectivity lower than 0.992, which is not a concern region and is therefore represented consistently in color. Our simulated results also indicate that tilting the incident angle of light will narrow the bandwidth for the reflectivity between 0.992 and 0.997. Furthermore, our simulations suggest that 17 pairs of NP GaN DBRs with an air voids ratio of *φ* = 0.6 would be an appropriate choice to achieve a reflective mirror with a high reflectivity of 0.997 under light incident angles between 0° and 20°, which can effectively suppress the reflection loss at high-order diffraction waves.

### 3.2. Optimization of TiO_2_ High-Index Contrast Grating

Similarly, the top high-reflectivity mirror is crucial in reducing thickness and providing specific polarization selectivity, especially for an InGaN-based VCSEL. Meanwhile, a monolithic GaN HCG formed on the n-GaN layer for blue VCSEL and was recently reported [36]. However, in our proposed VCSEL structure, a top n-GaN layer can serve as an etching stop layer for the high-index contrast grating of TiO_2_. This arrangement allows for easier control over the thickness of the cavity. In the simulation, the refractive index 2.54 of TiO_2_, which is higher than that of GaN, was adopted, and there was no absorption in the red light range, as reported previously [51].

To design the geometry of the TiO_2_ HCG optimally, forming a thin-film high-reflection mirror, two normalized grating structure variables, NW and NH, were employed. NW and NH used in the following simulation are defined as the TiO_2_ grating width and height simultaneously divided by the grating period. To efficiently identify TiO_2_ grating geometric parameters conducive to achieving a VCSEL emitting light at a wavelength of 612 nm, an optimization simulation model was established, ensuring that the reflectivity ratio of transverse electric/transverse magnetic (TM) is significantly greater than 1 to obtain a pure TE-polarized high-index contrast grating structure. This approach facilitates the rapid identification of satisfactory geometric parameters for optimal VCSEL performances, such as narrow linewidth, low lasing threshold, and polarization selectivity.

**Figure 3:**
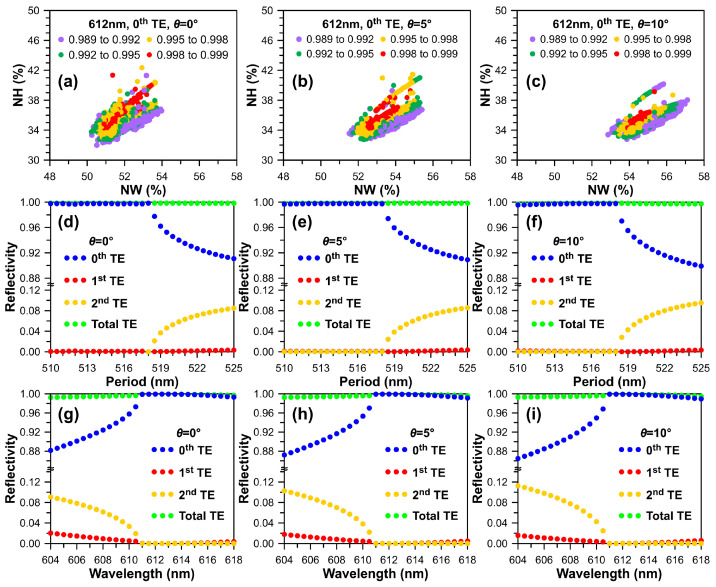
Simulated TE polarization reflectivities for optimized TiO_2_ HCGs are functions of normalized width (NW) and height (NH) for sidewall angles of (**a**) *θ* = 0°, (**b**) *θ* = 5°, and (**c**) *θ* = 10°. Period- and wavelength-dependent 0th, 1st, 2nd orders and total reflectivities for (**d**,**g**) *θ* = 0°, (**e**,**h**) *θ* = 5°, and (**f**,**i**) *θ* = 10°.

The optimally simulated TE-polarized reflectivities for the fast design of TiO_2_ HCGs with different values of NW and NH and sidewall angles of *θ* = 0°, *θ* = 5°, and *θ* = 10° are illustrated in Figure 3a–c. The disparate color dots represent the maximum TE-polarized reflectivity searching points, involving information on multiple sets of TiO_2_ grating periods and the optimized geometric parameters of TiO_2_ HCGs with a reflectivity of approximately 0.998 to 0.999 for the three sidewall angles. The optimized geometric parameters (NW, NH) are (51–54%, 34–40%), (52.5–55%, 34–38%), and (54–55.5%, 34–37%), respectively. Furthermore, a detailed analysis of period- and wavelength-dependent 0th, 1st, 2nd orders, and total reflectivities for TE-polarized light are presented in Figure 3d–i. As scattering parameters, also known as S-parameters, are the components of frequency-dependent complex-valued matrix and commonly used to calculate the transmittance and reflectance for zero-, first-, and high-order diffraction of electromagnetic waves, this approach has been widely adopted in various fields [52,53,54]. The total reflectivity is a combination of the reflectivity of 0th, 1st, and 2nd order reflections. It is observed from Figure 3d–f that although the total TE-polarization reflectivity remains close to unity, the zeroth-order reflectivity rapidly decreases when the TiO_2_ grating period exceeds 518 nm. In comparison, the incident light wavelength is fixed at 612 nm. 

Moreover, for a fixed TiO_2_ grating period of 517 nm, the optimized geometric parameters (51.36%, 35.21%), (52.85%, 35.24%), and (54.38%, 35.59%) are utilized for three sidewall angles to examine the wavelength-dependent TE-polarized reflectivities for different diffraction orders, as illustrated in Figure 3g–i. The simulated 0th order reflectivities can achieve meaningful values between 0.995 and 0.999 when the incident light wavelength is within the range of 611 nm to 618 nm. On the other hand, the influence of small sidewall angles can be disregarded.

### 3.3. Staggered Multiple Quantum Well Design

**Figure 4:**
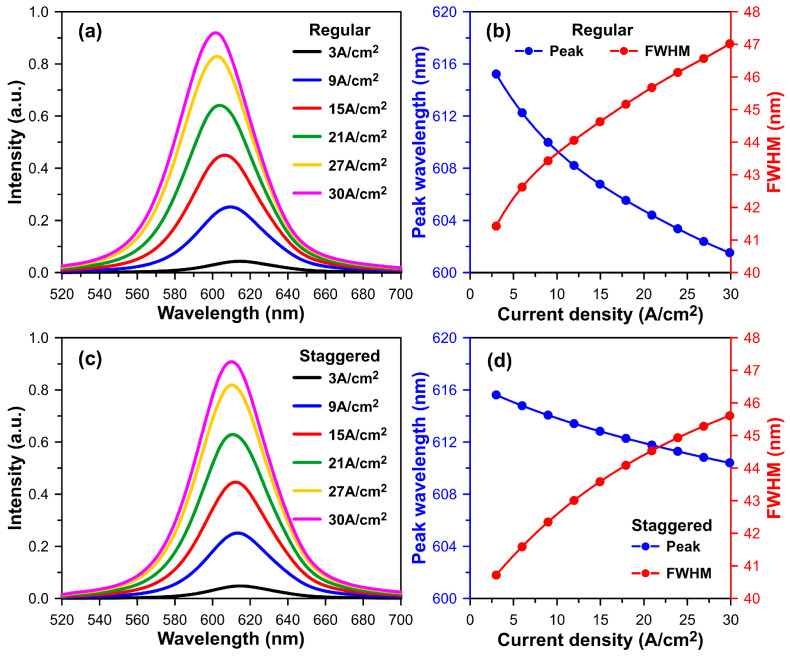
Simulated InGaN-based active region emission spectra with (**a**) regular and (**c**) staggered InGaN MQW designs. Correlated peak wavelengths and FWHMs as a function of current density for (**b**) regular and (**d**) staggered InGaN MQW designs.

In this subsection, we will conduct a numerical investigation of the optoelectronic characteristics of regular and staggered InGaN multiple quantum well designs. We will then briefly compare these two InGaN-based MQW designs. For the sake of simplicity, our emission spectrum simulation of the InGaN MQW structure still needs to consider the self-heating effect. As a result, Figure 4a,c display the simulated emission spectra of the optical active regions created by the regular (4 nm In_0.388_Ga_0.612_N) and symmetric staggered (1 nm In_0.217_Ga_0.783_N/2 nm In_0.434_Ga_0.566_N/1 nm In_0.217_Ga_0.783_N) MQW structure designs under an injection current density ranging from 3 A/cm^2^ to 30 A/cm^2^. Two types of InGaN MQWs with different Indium content were selected to emit light with a wavelength close to 615 nm when an injection current density of 3 A/cm^2^ was applied when the current aperture diameter was assumed to be 10 µm. In addition, the emission peak wavelengths and corresponding full width at half maximum of these two InGaN MQW designs for each current density are methodically listed in Figure 4b,d.

Drawing from previous studies [47,48] and our simulation results, we observe that the calculated emission peak wavelength of the regular InGaN MQW design shifts from 615.24 nm to 601.02 nm, resulting in a blue shift of 14.22 nm, and the calculated FWHM increases from 41.43 nm to 47.36 nm. Conversely, for the staggered InGaN MQW design, the emission peak wavelength and FWHM will shift from 615.62 nm to 610.18 nm and 40.72 nm to 45.76 nm, respectively. In the staggered MQW design used as the active region of an InGaN-based red VCSEL, the emission wavelength undergoes a relatively small blue shift of 5.44 nm when the injection current density increases from 3 A/cm^2^ to 30 A/cm^2^. Furthermore, calculated FWHMs of regular and staggered MQW designs, which are initially more than 40 nm, are further narrowed by the resonant cavity effect. When the emission wavelength of the InGaN MQW aligns with the laser mode wavelength of the Fabry–Pérot (F-P) like cavity, the light output efficiency can be significantly enhanced, and the FWHM can be greatly reduced. These predictions were based on the findings of a previous study on green µ-RCLED [48].

### 3.4. Cavity Mode Wavelength of VCSEL Unit Cell Model

**Figure 5:**
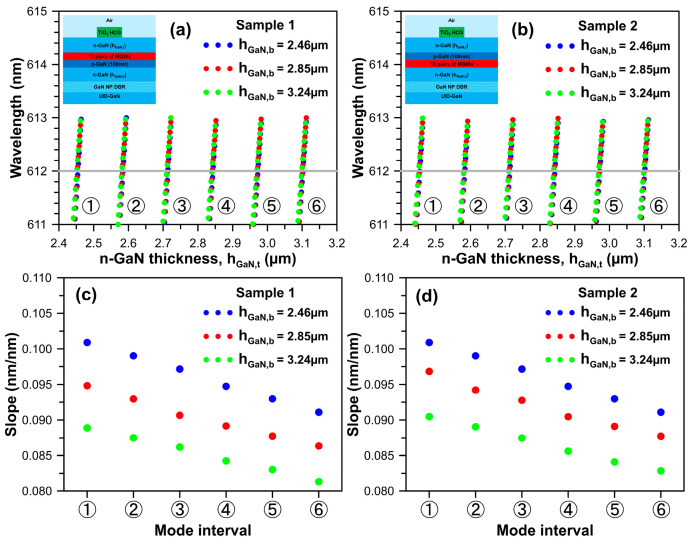
Calculated cavity mode wavelengths of proposed InGaN-based red VCSEL unit cell varying with n-GaN thickness, h_GaN,t_ for (**a**) simulation sample 1: VCSEL with n-p-i-n epi-structure design and (**b**) simulation sample 2: VCSEL with n-i-p-n epi-structure design. The insets are schematics of two samples. (**c**,**d**) indicate the wavelength slopes for six mode intervals found in (**a**,**b**). The slope means the n-GaN thickness varies every 1 nm; the longitudinal mode wavelength will be blue- or red-shifted from 0.08 nm to 0.1 nm. The gray lines indicated the target wavelength of 612 nm.

Here, we will examine the impact of n-GaN thickness on the F-P-like longitudinal mode wavelengths of the InGaN-based red vertical-cavity surface-emitting lasers constructed by combining 17 pairs of nanoporous n-doped GaN/ undoped GaN DBR, 10 pairs of staggered InGaN MQWs, and TiO_2_ high-index contrast grating structure with a period of 517 nm, a width of 270.1 nm, a height of 182.2 nm, and a sidewall angle *θ* = 5°. To briefly discuss the thickness effect of the n-GaN layer in the longitudinal mode wavelength and optical threshold gain, the VCSEL unit cell models for simulation sample 1 (n-p-i-n structure) and simulation sample 2 (n-i-p-n structure) are employed, and corresponding design schematics of the two epi-structures are depicted in the insets of Figure 5a and 5b, respectively.

As is commonly understood, an appropriate cavity length can enhance heat dissipation to prevent a significant redshift of the longitudinal mode caused by the thermo-optic effect, given that the thermo-optic coefficient, dn/dT, for GaN is on the order of 10^−4^ [55]. Accordingly, the cavity mode wavelengths of the proposed InGaN-based red vertical-cavity surface-emitting lasers are calculated by varying the top n-GaN thickness, h_GaN,t_, using the unit cell models. In Figure 5a,b, three different bottom n-GaN thicknesses, h_GaN,b_ = 2.46 µm, 2.85 µm, and 3.24 µm, are considered, corresponding to 9.5, 11.0, and 12.5 times the wavelength of light, respectively. Each colored dot in Figure 5 represents different orders of longitudinal Fabry–Perot modes. Six numbered circles are associated with six mode intervals for top n-GaN thicknesses changing from 2.4 µm to 3.2 µm. The calculated spacing between adjacent repeat mode intervals is approximately 130 nm.

Additionally, the calculated slopes of cavity mode wavelengths for the six mode intervals of n-p-i-n and n-i-p-n structures are illustrated in Figure 5c,d. The slope indicates that for every 1 nm variation in top n-GaN thickness, the longitudinal mode wavelength experiences a blue- or redshift of 0.08 nm to 0.1 nm. Our calculations indicate that a slightly smaller mode wavelength slope for a VCSEL with a thicker top n-GaN layer can be observed. Furthermore, in cases of three symmetrical n-GaN layer arrangements, the following three thicknesses are selected: (1) h_GaN,t_(=h_GaN,b_) = 2.46 µm, (2) h_GaN,t_(=h_GaN,b_) = 2.85 µm, and (3) h_GaN,t_(=h_GaN,b_) = 3.24 µm. The simulated results revealed that the mode spacings near the target wavelength of 612 nm at approximately 13.7 nm, 11.9 nm, and 10.8 nm, respectively. It is evident from this simulation analysis that when the total n-GaN layer thickness, h_GaN,t_ + h_GaN,b_, is less than or equal to 6.48 µm, the longitudinal mode spacing will be equal to and more significant than 10 nm.

### 3.5. Threshold Gain of Finite VCSEL Model

In this section, the HCG pattern size can be treated as the optical aperture size. After the simulation results outlined above, the cavity mode wavelengths and threshold gains of the two-dimensional (2D) InGaN-based red VCSEL model with finite periods of TiO_2_ high-index contrast gratings will be examined. Initially, the thicknesses of the top and bottom n-GaN layers were set to h_GaN,t_ = 2.72 µm and h_GaN,b_ = 2.85 µm, which serve as an illustrative simulation example. 

**Figure 6:**
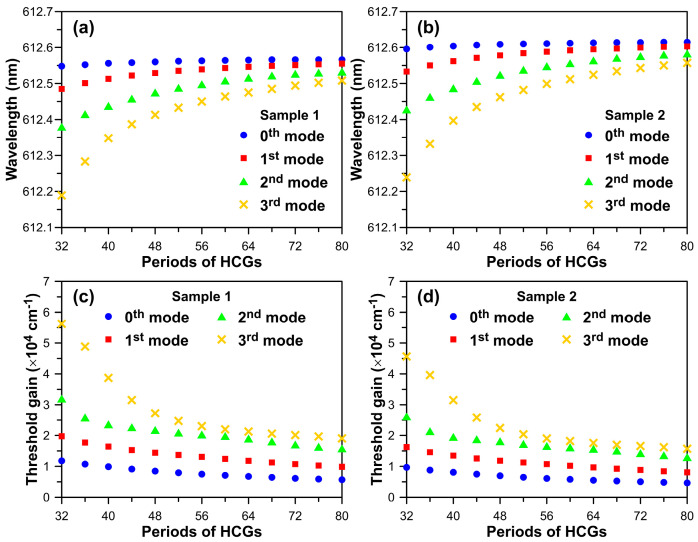
The calculated F-P cavity mode wavelengths and threshold gains of InGaN-based red VCSELs varying with finite periods of TiO_2_ gratings for (**a**,**c**) VCSEL with n-p-i-n epi-structure design and (**b**,**d**) VCSEL with n-i-p-n epi-structure design. Here, top and bottom n-GaN thicknesses are assumed to be h_GaN,t_ = 2.72 μm and h_GaN,b_ = 2.85 μm. The four kinds of symbols express the calculated 0th to 3rd orders transverse modes.

The F-P cavity mode wavelengths (threshold gains) for InGaN-based red VCSEL unit cells with h_GaN,t_ = 2.72 µm and h_GaN,b_ = 2.85 µm for n-p-i-n epi-structure and n-i-p-n epi-structure designs are simulated as follows: 612.73 nm (4003.6 cm^−1^) and 612.77 nm (3304 cm^−1^). Simulation sample 1 exhibits a higher threshold gain, primarily due to the small optical confinement factor inside the MQW. A comparison of the calculated cavity mode wavelengths and threshold gains of the 2D finite VCSEL model with different optical aperture sizes for n-p-i-n epi-structure and n-i-p-n epi-structure are presented in Figure 6. The period number of TiO_2_ grating varies from 32 to 80, corresponding to changes in the optical aperture size from 16.54 µm to 41.36 µm. Consequently, the calculated mode wavelength and threshold gain will rapidly increase and decrease with an increase in the TiO_2_ grating periods, especially for high-order transverse modes. The desired cavity mode wavelengths of simulation samples 1 and 2 will approach a constant value at 612.56 nm and 612.62 nm, respectively, when the period of TiO_2_ grating reaches 80. The wavelength discrepancy between the 0th (symbolized by a blue solid circle) and 3rd (symbolized by a yellow cross) order modes reduces from 0.36 nm to 0.06 nm for both VCSEL epi-structure designs.

Furthermore, as the period number of TiO_2_ high-index contrast gratings broadens from 32 to 80, the differences in calculated threshold gain between the fundamental and first-order transverse modes will reduce from 7942 cm^−1^ to 4174 cm^−1^ for VCSEL with n-p-i-n epi-structure design and 6569 cm^−1^ to 3440 cm^−1^ for VCSEL with n-i-p-n epi-structure design, respectively. The threshold gains of the first-order mode for the two designed epi-structures increased by 67.4–73.3% and 67.9–74.0%, relative to the fundamental mode. It is evident that lateral scattering loss strongly influences the threshold gain of the fundamental mode for the VCSEL with a smaller amount of high-index contrast grating. Notably, the calculated threshold gain of the VCSEL with an n-p-i-n epi-structure will increase by approximately 21.8 ± 0.3% compared to that of the VCSEL with an n-i-p-n epi-structure for each transverse mode. This value is very close to the threshold increment ratio of 21.2%, as evaluated from the results of the two types of VCSEL unit cell models. The aforementioned explanations suggest that the proposed VCSEL unit cell model is a simple and fast model for exploring the optimal geometric parameters of VCSELs with a low lasing threshold. Ultimately, the comprehensive simulated results of this study will contribute to the further development of high-polarization and high-brightness red light sources.

## 4. Conclusions

We have proposed and conducted numerical investigations into the optoelectronic characteristics of an InGaN-based red vertical-cavity surface-emitting laser constructed with a p-n junction, staggered InGaN MQW, NP GaN DBR, and TiO_2_ HCG. We systematically and numerically explored the effects of geometric parameters for the NP GaN DBR and TiO_2_ HCG, including pair number, air voids ratio, incident angle, width, height, sidewall angle, and finite period effect of the HCG pattern. Our calculations revealed that the reflectance of 17 pairs of NP GaN DBRs with an air voids ratio of φ = 0.6 can be higher than 99.7%, which is a suitable option for creating a high-reflectivity mirror under a light incident angle between 0° and 20°. Additionally, the zeroth-order reflectivity of TiO_2_ HCG rapidly decreases when the period of the HCG is more prominent than 518 nm for a light wavelength of 612 nm, and the normalized geometric parameters NW and NH of the TiO_2_ HCG are optimized. The average optimal geometric parameters for an HCG period of 517 nm are width-to-period (52.86 ± 1.5%) and height-to-period (35.35 ± 0.14%) when the sidewall angle is within the range of 0° to 10°. Furthermore, in comparison to a conventional InGaN MQW design composed of three pairs of square potential wells, the simulated emission wavelength has a relatively small blue shift of 5.44 nm for the active region made by the symmetric staggered InGaN MQW design when the injection current density increases from 3 A/cm^2^ to 30 A/cm^2^. Furthermore, the calculation results obtained from our proposed VCSEL unit cell model made the following predictions: (1) the desired cavity mode wavelength repeats every 130 nm as the n-GaN thickness changes, (2) the cavity mode wavelength will shift by 0.08 nm to 0.1 nm for every 1 nm change in n-GaN thickness, and (3) if the total n-GaN thickness is less than or equal to 6.48 µm, the longitudinal mode spacing will be larger than 10 nm. This proposed VCSEL unit cell model in this study is simple and efficient for exploring the optimal geometric parameters. Moreover, we also investigated the longitudinal mode wavelengths and corresponding optical threshold gains of the 2D finite VCSEL model with limited periods of TiO_2_ HCGs. The simulated results demonstrated that the longitudinal mode wavelength and threshold gain will rapidly increase and decrease with the HCG pattern size manipulation as the period number of the TiO_2_ grating varies from 32 to 80. For our proposed VCSELs with n-p-i-n and n-i-p-n epi-structure designs, the difference in cavity wavelength between the 0th and 3rd orders of transverse modes is only 0.36 nm to 0.06 nm. However, the differences in threshold gain between the 0th and 1st order transverse modes will decrease from 7942 cm^−1^ to 4174 cm^−1^ for the VCSEL with an n-p-i-n epi-structure and 6569 cm^−1^ to 3440 cm^−1^ for the VCSEL with an n-i-p-n epi-structure. A significant threshold gain difference of approximately 67.4–74% between the 0th and 1st order transverse modes can be obtained. Due to the fewer TiO_2_ grating periods, the threshold of the fundamental transverse mode is dominated by lateral scattering loss. In summary, our simulation results demonstrate that the proposed InGaN-based red VCSELs can serve as a foundational principle for further investigations of next-generation light sources.

## Data Availability

Data are contained within the article.

## References

[1] Chellappan K., Erden V.E., Urey H. (2010). Laser-based displays: A review. Appl. Opt..

[2] Iga K. (2021). Appendix G: Laser Displays and TV. VCSEL Industry: Communication and Sensing.

[3] Rodes R., Müeller M., Li B., Estaran J., Jensen J.B., Gruendl T., Ortsiefer M., Neumeyr C., Rosskopf J., Larsen K.J. (2013). High-speed 1550 nm VCSEL data transmission link employing 25 GBd 4-PAM modulation and hard decision forward error correction. J. Light. Technol..

[4] Lee M.W., Hong Y., Shore K.A. (2004). Experimental demonstration of VCSEL-based chaotic optical communications. IEEE Photon. Technol. Lett..

[5] Hsiao F.H., Lee T.Y., Miao W.C., Pai Y.H., Iida D., Lin C.L., Chen F.C., Chow C.W., Lin C.C., Horng R.H. (2023). Investigations on the high performance of InGaN red micro-LEDs with single quantum well for visible light communication applications. Discover Nano.

[6] Shin H.-K., Kim I., Kim E.-J., Kim J.-H., Lee E.-K., Lee M.-K., Mun J.-K., Park C.-S., Yi Y.-S. (1996). Vertical-Cavity Surface-Emitting Lasers for Optical Data Storage. Jpn. J. Appl. Phys..

[7] Martin Y., Rishton S., Wickramasinghe H.K. (1997). Optical data storage read out at 256 Gbits/in.^2^. Appl. Phys. Lett..

[8] Khan A., Schaefer D., Tao L., Miller D.J., Sun K., Zondlo M.A., Harrison W.A., Roscoe B., Lary D.J. (2012). Power greenhouse gas sensors for unmanned aerial vehicles. Remote Sens..

[9] Lan L., Chen J., Zhao X., Ghasemifard H. (2019). VCSEL-based atmospheric trace gas sensor using first harmonic detection. IEEE Sens. J..

[10] Sangu S., Shimokawa T., Tanaka S. Ultracompact eye and pupil tracking device using VCSEL arrays and position sensitive detector. Proceedings of the SPIE, Optical Architectures for Displays and Sensing in Augmented, Virtual, and Mixed Reality (AR, VR, MR), 113101F.

[11] Khaldi A., Daniel E., Massin L., Kärnfelt C., Ferranti F., Lahuec C., Seguin F., Nourrit V., de Bougrenet de la Tocnaye J.-L. (2020). A laser emitting contact lens for eye tracking. Sci. Rep..

[12] Fujioka I., Ho Z., Gu X., Koyama F. Solid State LiDAR with Sensing Distance of over 40 m using a VCSEL Beam Scanner. Proceedings of the Conference on Lasers and Electro-Optics (CLEO).

[13] Dummer M., Johnson K., Rothwell S., Tatah K., Hibbs-Brenner M. The role of VCSELs in 3D sensing and LiDAR. Proceedings of the SPIE, Optical Interconnects XXI, 116920C.

[14] Liu A., Wolf P., Lott J.A., Bimberg D. (2019). Vertical-cavity surface-emitting lasers for data communication and sensing. Photonics Res..

[15] Han Y., Li Z., Wu L., Mai S., Xing X., Fu H.Y. (2023). High-Speed Two-Dimensional Spectral-Scanning Coherent LiDAR System Based on Tunable VCSEL. J. Light. Technol..

[16] Chen B., Claus D., Russ D., Nizami M.R. (2020). Generation of a high-resolution 3D-printed freeform collimator for VCSEL-based 3D-depth sensing. Opt. Lett..

[17] Hamaguchi T., Tanaka M., Mitomo J., Nakajima H., Ito M., Ohara M., Kobayashi N., Fujii K., Watanabe H., Satou S. (2018). Lateral optical confinement of GaN-based VCSEL using an atomically smooth monolithic curved mirror. Sci. Rep..

[18] Lee S.G., Forman C.A., Kearns J., Leonard J.T., Cohen D.A., Nakamura S., DenBaars S.P. (2019). Demonstration of GaN-based vertical-cavity surface-emitting lasers with buried tunnel junction contacts. Opt. Express.

[19] Chang T.C., Hong K.B., Kuo S.K., Lu T.C. (2019). Demonstration of polarization control GaN-based micro-cavity lasers using a rigid high-contrast grating reflector. Sci. Rep..

[20] Zheng Z., Mei Y., Long H., Hoo J., Guo S., Li Q., Ying L., Zheng Z., Zhang B. (2021). AlGaN-based deep ultraviolet vertical-cavity surface-emitting laser. IEEE Electron Device Lett..

[21] Hjort F., Enslin J., Cobet M., Bergmann M.A., Gustavsson J., Kolbe T., Knauer A., Nippert F., Häusler I., Wagner M.R. (2021). A 310 nm Optically Pumped AlGaN Vertical-Cavity Surface-Emitting Laser. ACS Photonics.

[22] Chen C.C., Liaw S.J., Yang Y.J. (2001). Stable Single-Mode Operation of an 850-nm VCSEL with a Higher Order Mode Absorber Formed by Shallow Zn Diffusion. IEEE Photon. Technol. Lett..

[23] Holder C.O., Leonard J.T., Farrell R.M., Cohen D.A., Yonkee B., Speck J.S., DenBaars S.P., Nakamura S., Feezell D.F. (2014). Nonpolar III-nitride vertical-cavity surface emitting lasers with a polarization ratio of 100% fabricated using photoelectrochemical etching. Appl. Phys. Lett..

[24] Kuramoto M., Kobayashi S., Akagi T., Tazawa K., Tanaka K., Saito T., Takeuchi T. (2018). Enhancement of slope efficiency and output power in GaN-based vertical-cavity surface-emitting lasers with a SiO_2_-buried lateral index guide. Appl. Phys. Lett..

[25] Leonard J.T., Young E.C., Yonkee B.P., Cohen D.A., Margalith T., DenBaars S.P., Speck J.S., Nakamura S. (2015). Demonstration of a III-nitride vertical-cavity surface-emitting laser with a III-nitride tunnel junction intracavity contact. Appl. Phys. Lett..

[26] Hamaguchi T., Tanaka M., Nakajima H. (2019). A review on the latest progress of visible GaNbased VCSELs with lateral confinement by curved dielectric DBR reflector and boron ion implantation. Jpn. J. Appl. Phys..

[27] Wang Y.-C., Yang T., Shi L., Chen Y.-H., Mei Y., Zhang B.-P. (2023). Simulation of performance enhancement of GaN-based VCSELs by composition gradient InGaN last-quantum barrier. Semicond. Sci. Technol..

[28] Mishkat-Ul-Masabih S.M., Aragon A.A., Monavarian M., Luk T.S., Feezell D.F. (2019). Electrically injected nonpolar GaN-based VCSELs with lattice-matched nanoporous distributed Bragg reflector mirrors. Appl. Phys. Express.

[29] Elafandy R.T., Kang J.-H., Mi C., Kim T.K., Kwak J.S., Han J. (2021). Study and Application of Birefringent Nanoporous GaN in the Polarization Control of Blue Vertical-Cavity Surface-Emitting Lasers. ACS Photonics.

[30] Horng R.-H., Ye C.-X., Chen P.-W., Iida D., Ohkawa K., Wu Y.-R., Wuu D.-S. (2022). Study on the effect of size on InGaN red micro-LEDs. Sci. Rep..

[31] Chang-Hasnain C.J., Zhou Y., Huang M.C.Y., Chase C. (2009). High-contrast grating VCSELs. IEEE J. Sel. Top. Quantum Electron..

[32] Chang-Hasnain C.J., Yang W. (2012). High-contrast gratings for integrated optoelectronics. Adv. Opt. Photonics.

[33] Kim S., Wang Z., Brodbeck S., Schneider C., Höfling S., Deng H. (2019). Monolithic high-contrast grating based polariton laser. ACS Photonics.

[34] Gębski M., Lott J.A., Czyszanowski T. (2019). Electrically injected VCSEL with a composite DBR and MHCG reflector. Opt. Express.

[35] Haglund Å., Hashemi E., Bengtsson J., Gustavsson J., Stattin M., Calciati M., Goano M. Progress and challenges in electrically pumped GaN-based VCSELs. Proceedings of the SPIE 9892, Semiconductor Lasers and Laser Dynamics VII, 98920Y.

[36] Hong K.-B., Chang T.-C., Hjort F., Lindvall N., Hsieh W.-H., Huang W.-H., Tsai P.-H., Czyszanowski T., Haglund Å., Lu T.-C. (2021). Monolithic high-index contrast grating mirror for a GaN-based vertical-cavity surface-emitting laser. Photon. Res..

[37] Czyszanowski T., Gebski M., Dems M., Wasiak M., Sarzała R., Panajotov K. (2017). Subwavelength grating as both emission mirror and electrical contact for VCSELs in any material system. Sci. Rep..

[38] Hu H., Zhou S., Wan H., Liu X., Li N., Xu H. (2019). Effect of strain relaxation on performance of InGaN/GaN green LEDs grown on 4-inch sapphire substrate with sputtered AlN nucleation layer. Sci. Rep..

[39] Zhuang Z., Iida D., Velazquez-Rizo M., Ohkawa K. (2021). 606-nm InGaN amber micro-light-emitting diodes with an on-wafer external quantum efficiency of 0.56%. IEEE Electron Device Lett..

[40] Zhuang Z., Iida D., Ohkawa K. (2021). Investigation of InGaN-based red/green micro-light-emitting diodes. Opt. Lett..

[41] Arif R.A., Zhao H., Ee Y.K., Tansu N. (2008). Spontaneous emission and characteristics of staggered InGaN quantum-well light-emitting diodes. IEEE J. Quantum Electron..

[42] Zhao H., Arif R.A., Tansu N. (2009). Design analysis of staggered InGaN quantum wells light-emitting diodes at 500–540 nm. IEEE J. Sel. Top. Quantum Electron..

[43] Zhang Z.H., Liu W., Ju Z.G., Tan S.T., Ji Y., Kyaw Z., Zhang X.L., Wang L.C., Sun X.W., Demir H.V. (2014). Self-screening of the quantum confined stark effect by the polarization induced bulk charges in the quantum barriers. Appl. Phys. Lett..

[44] Gao Y., Chu C., Hang S., Zhang Y., Zhang Z.-H., Zhou J. (2021). Quantum barriers with a polarization self-screening effect for GaN-based VCSELs to increase the electron-hole stimulated recombination and output performance. Opt. Mater. Express.

[45] Cai L.-E., Xu C.-Z., Xiong F.-B., Zhao M.-J., Lin H.-F., Lin H.-Y., Sun D. (2021). Improved carrier confinement and distribution in InGaN light-emitting diodes with three-layer staggered QWs. AIP Adv..

[46] Usman M., Munsif M., Anwar A.-R., Mushtaq U., Imtiaz W.A., Han D.-P., Muhammad N. (2019). Zigzag-shaped quantum well engineering of green light-emitting diode. Superlattices Microstruct..

[47] Gladysiewicz M., Hommel D., Kudrawiec R. (2019). Material Gain Engineering in Staggered Polar AlGaN/AlN Quantum Wells Dedicated for Deep UV Lasers. IEEE J. Sel. Top. Quantum Electron..

[48] Hsieh T.-H., Huang W.-T., Hong K.-B., Lee T.-Y., Bai Y.-H., Pai Y.-H., Tu C.-C., Huang C.-H., Li Y., Kuo H.-C. (2023). Optoelectronic Simulations of InGaN-Based Green Micro-Resonant Cavity Light-Emitting Diodes with Staggered Multiple Quantum Wells. Crystals.

[49] Kivisaari P., Kim I., Suihkonen S., Oksanen J. (2017). Elimination of lateral resistance and current crowding in large-area LEDs by composition grading and diffusion-driven charge transport. Adv. Electron. Mater..

[50] Lee M.L., Wang S.S., Yeh Y.H., Liao P.H., Sheu J.K. (2019). Light-emitting diodes with surface gallium nitride p–n homojunction structure formed by selective area regrowth. Sci. Rep..

[51] Davis K.O., Jiang K., Habermann D., Schoenfeld W.V. (2015). Tailoring the Optical Properties of APCVD Titanium Oxide Films for All-Oxide Multilayer Antireflection Coatings. IEEE J. Photovolt..

[52] Sharma A.K., Pandey A.K. (2019). Self-referenced plasmonic sensor with TiO_2_ grating on thin Au layer: Simulated performance analysis in optical communication band. J. Opt. Soc. Am. B.

[53] Algorri J.F., Morawiak P., Zografopoulos D.C., Bennis N., Spadlo A., Rodríguez-Cobo L., Jaroszewicz L.R., Sánchez-Pena J.M., López-Higuera J.M. (2020). Multifunctional light beam control device by stimuli-responsive liquid crystal micro-grating structures. Sci. Rep..

[54] Hu Y., Zhang Y., Su G., Zhao M., Li B., Liu Y., Li Z. (2022). Realization of ultrathin waveguides by elastic metagratings. Commun. Phys..

[55] Zhao G.Y., Ishikawa H., Yu G., Egawa T., Watanabe J., Soga T., Jimbo T., Umeno M. (1998). Thermo-optical nonlinearity of GaN grown by metalorganic chemical vapor deposition. Appl. Phys. Lett..

